# Supplementary material for: An enhanced split intein-mediated ligation (SIML) platform for rapid discovery and functional screening of circular bacteriocins
Source: Front Bioeng Biotechnol. 2026 Sep 24;14:1950086. doi: 10.3389/fbioe.2026.1950086 (PMC13646528; doi:10.3389/fbioe.2026.1950086)
Supplement: Supplementary file 1 [file DataSheet1.pdf]

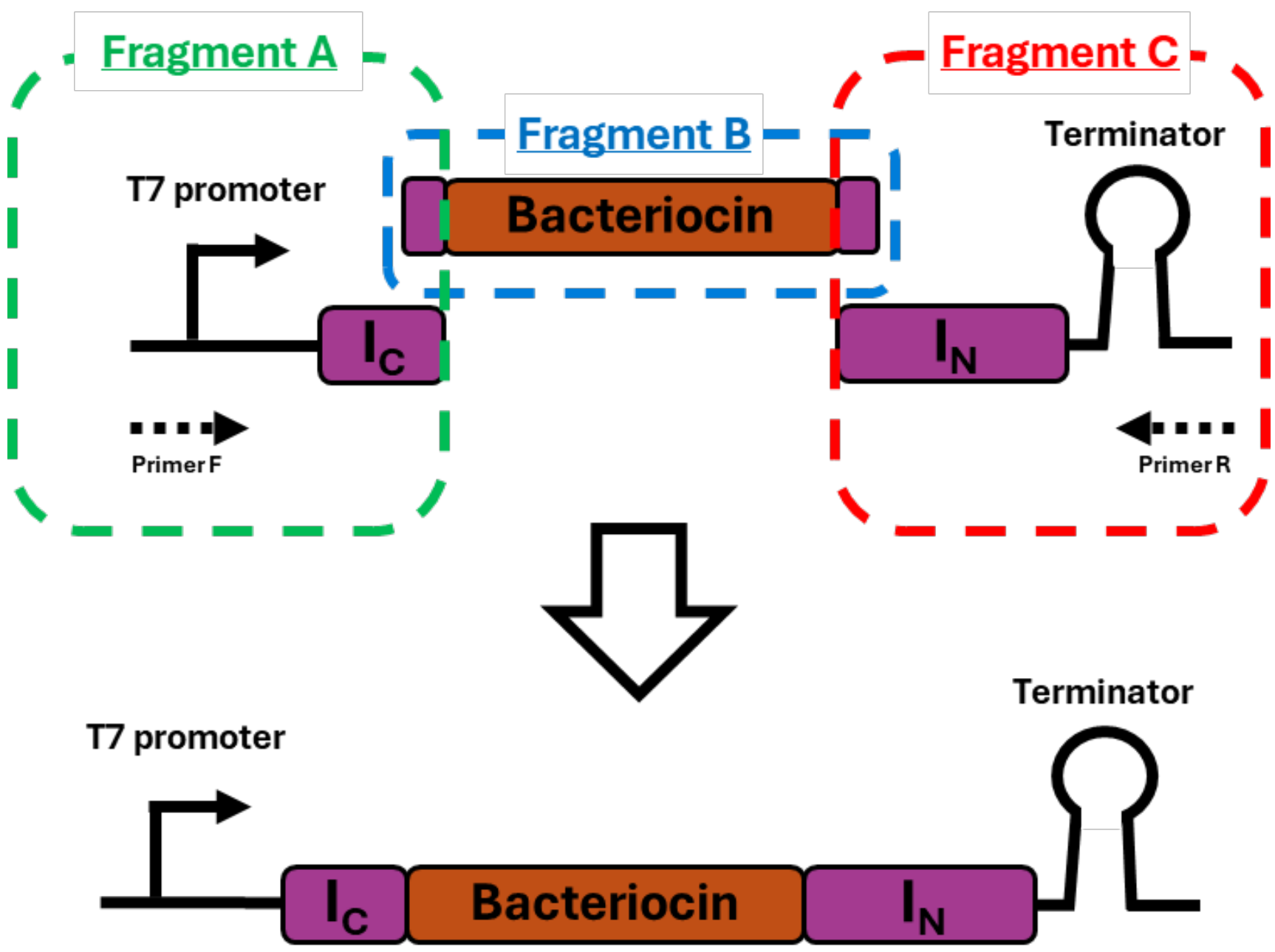

Figure S1. Schematic representation of SIML expression cassette assembly by overlap-extension PCR. The SIML constructs were generated using a three-fragment assembly strategy. **Fragment A** contains the T7 promoter followed by the C-terminal fragment of the split intein (I_C). **Fragment B** encodes the mature circular bacteriocin sequence, beginning at the selected serine residue used as the +1 nucleophile for cyclization, and is flanked by regions homologous to I_C at the 5′ end and to the N-terminal intein fragment (I_N) at the 3′ end. **Fragment C** contains the I_N fragment followed by a transcription terminator. The three fragments were assembled by overlap-extension PCR to generate a complete T7–I_C–bacteriocin–I_N–terminator expression cassette, which was purified and used directly as a DNA template for *in vitro* cell-free protein synthesis (IV-CFPS) reactions within the SIML framework.

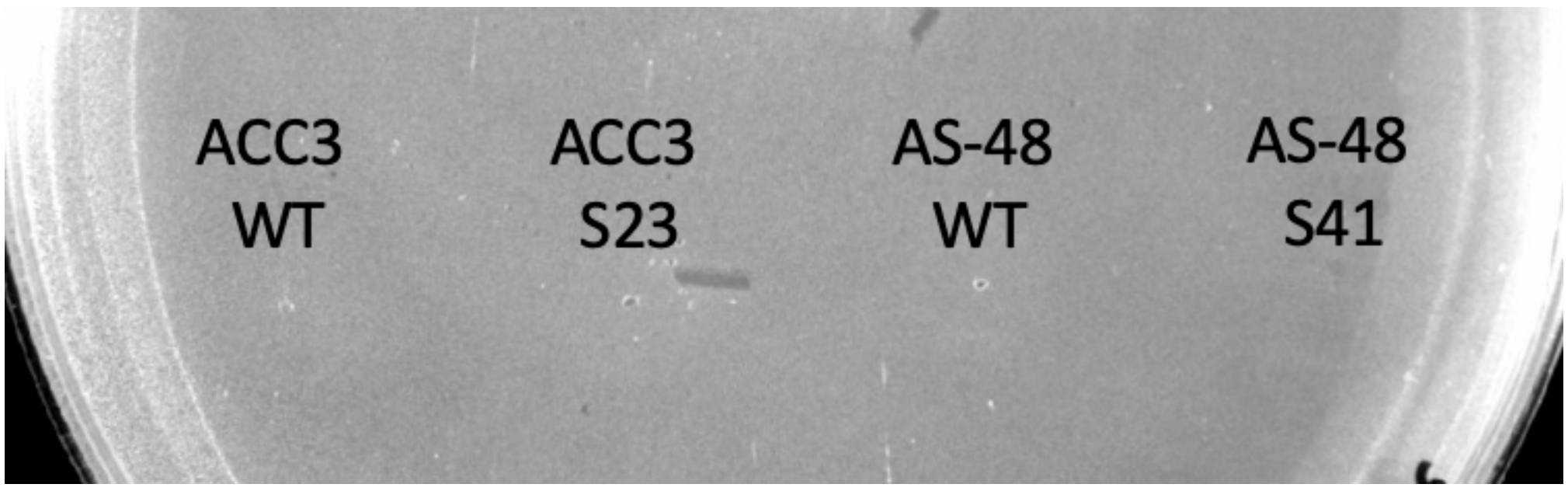

Figure S2. Antimicrobial activity of intein-free EntAS-48 and AmCyc constructs produced by IV-CFPS. For each bacteriocin, two linear constructs lacking the split intein components were evaluated: one starting at the native N-terminal residue (WT) and one starting at the serine residue corresponding to the cyclization site of the selected SIML construct (S41 for EntAS-48 and S23 for AmCyc). No detectable antimicrobial activity was observed for any of the intein-free constructs.

Table S1. Nucleotide and amino acid sequences used for SIML construct design and circular bacteriocin variant libraries. The table summarizes the DNA and protein sequences employed in the generation of SIML expression cassettes. The upper section lists the nucleotide sequences of the T7 promoter, transcription terminator, and primers used for cassette assembly. The middle section shows the amino acid sequences of the split inteins used in this study, including NpuDnaE, Gp41-1, and SspGyrB, with their corresponding C-terminal (I_C) and N-terminal (I_N) fragments. The lower section details the amino acid sequences of the circular bacteriocins and their variants evaluated.

	Nucleotidic sequence (5´- 3´)
**T7 promoter**	GCGAATTAATACGACTCACTATAGGGCTTAAGTATAAGGAGGAAAAAAT
**Terminator**	TAACTAGCATAACCCCTCTCTAAACGGAGGGGTTT
**Primer F**	GCGAATTAATACGACTCACTATAG
**Primer R**	AAACCCCTCCGTTTAGAG

Split intein	Amino acid sequence
**NpuDnaE I_C**	MIKIATRKYLGKQNVYDIGVERYHNFALKNGFIASN
**NpuDnaE I_N**	CLSYDTEILTVEYGILPIGKIVEKRIECTVYSVDNNGNIYTQPVAQWHDRGEQEVFEYCLEDGCLIRATK DHKFMTVDGQMMPIDEIFERELDLMRVDNLPNGT
**Gp41-1 I_C**	MMLKKILKIEELDERELIDIEVSGNHLFYANDILTHN
**Gp41-1 I_N**	CLDLKTQVQTPQGMKEISNIQVGDLVLSNTGYNEVLNVFPKSKKKSYKITLEDGKEIICSEEHLFPTQTG EMNISGGLKEGMCLYVKE
**SspGyrB I_C**	MEAVLNYNHRIVNIEAVSETIDVYDIEVPHTHNFALASGVFVHN
**SspGyrB I_N**	CFSGDTLVALTDGRSVSFEQLVEEEKQGKQNFCYTIRHDGSIGVEKIINARKTKTNAKVIKVTLDNGESII CTPDHKFMLRDGSYKCAMDLTLDDSLMPLHRKISTTEDSGHA

Bacteriocin	Amino acid sequence
**AS48_S30**	SILTAVGSGGLSLLAAAGRESIKAYLKKEIKKKGKRAVIAWMAKEFGIPAAVAGTVLNVVEAGGWVTTIV
**AS48_S37**	SGGLSLLAAAGRESIKAYLKKEIKKKGKRAVIAWMAKEFGIPAAVAGTVLNVVEAGGWVTTIVSILTAVG
**AS48_S41 (AS48_1)**	SLLAAAGRESIKAYLKKEIKKKGKRAVIAWMAKEFGIPAAVAGTVLNVVEAGGWVTTIVSILTAVGSGGL
**AS48_S50**	SIKAYLKKEIKKKGKRAVIAWMAKEFGIPAAVAGTVLNVVEAGGWVTTIVSILTAVGSGGLSLLAAAGRE
**GarML_S19**	SAGMDIATALSLFSGAFTAAGGIMALIKKYAQKKLWKQLIAALVATGMAAGVAKTIVNAV
**GarML_S29 (GarML_1)**	SLFSGAFTAAGGIMALIKKYAQKKLWKQLIAALVATGMAAGVAKTIVNAVSAGMDIATAL
**GarML_S32**	SGAFTAAGGIMALIKKYAQKKLWKQLIAALVATGMAAGVAKTIVNAVSAGMDIATALSLF
**ACC_S3**	STLGISTAAAKKAIDIIDAASTIASIISLIGIVTGAGAISYAIVATAKTMIKKYGKKYAAAWLA
**ACC_S8**	STAAAKKAIDIIDAASTIASIISLIGIVTGAGAISYAIVATAKTMIKKYGKKYAAAWLASTLGI
**ACC_S23 (ACC_1)**	STIASIISLIGIVTGAGAISYAIVATAKTMIKKYGKKYAAAWLASTLGISTAAAKKAIDIIDAA
**ACC_S30**	SLIGIVTGAGAISYAIVATAKTMIKKYGKKYAAAWLASTLGISTAAAKKAIDIIDAASTIASII
**AS48_2**	SLIAAAGKESIKVYLKKQIKKKGRKAVIAWLAKEFGIPGGVAATVLNIVEAGGWATTILSILTAVGSGGL
**AS48_3**	SLIAAAGKETIRQYLKNEIKKKGRKAVIAWLAKEFGIPGSVAAVVLNVVEAGGAVTTIVSILTAVGSGGL
**AS48_4**	SLLAAAGKETIRQFLKKKIQEKGKRATIAWLAKEFGIPASIAAVVLNIVNAGGAVTTIVSILLAVGSGGL
**AS48_5**	SLIAAAGKETIRQYLRKKIEEKGRKAVIAWLAKEFGIPGGVASTVVWLAEAGGAAATIVGILAALGTGGL
**AS48_6**	SLIYAAGRQTIKAYLRREIRKRGRKAVIAWLVKEFGIPAAGASGLLWYLDAGSSAATIVGFLTGLASGGL
**AS48_7**	SLIAAAGTMGIKKYLKEELEKRGKQAFIAWLAKEFGVPAWAAAWILTALDASTTVTAIVSFLTALGTGGL
**AS48_8**	SLLAAAGKESLKAFLKKKYGEMGARAFTAWMVKEFGVPVAVATTVAKIMEAADWLITGAMILAAIGTGGA
**AS48_9**	SLIAAAGKLSIKAFLNKKKRELGTRAFVAWLMKEFGLPSVAAYTILYYLNVGSTVTTIISILTGLATGGL
**AS48_10**	SLVAAAEGQALRAFIKSQIRKIGKKAVIAWLAKEFAIPSTAAVIAVDVIDNGMTLVSVAAVITAIGTGGA
**GarML_2**	SLFSGAFTGVAGIVALLRKYAQRKLWQRFIAALVATGMSYTMARGIANAVSMGMNIWTAV

**GarML_3**	SMFAGVAGAGAFVLANGLKWITKWAGKQTIINYLVAAGIGAGNAVAIINAIDNGMNIVTAL
**GarML_4**	SLYAGLAGGAAWFLENGLKTLIKWAGKRTIISWLVATGMGAGTAASVVDAIFGGLTIASAL
**GarML_5**	SLYAGLAGGAAWFLEKGLTSLIRWAGRETIISWLVATGMAAGTASTIVNAILNGMDIAAAL
**ACC_2**	STIASIISLIGIVTGAGAVSYAIVATAKTMIKKYGKKYAAAWLASTLGISTAAAKKAIDIIDAA
**ACC_3**	STVASIISLIGIVTGAGAISYAIVATAKSMIKKYGKKYAAAWLASTLGISTAAAKKAIDIIDAA
**ACC_4**	STIASIISLIGVVTGAGAISYAVVATAKAMIKKYGKKYAAAWLASTLGISTAAAKKAIDIIDTA
**ACC_5**	STVATIISLATAVIGAGAISAAIVATAKKMIKKYGKKYAAAWLASTLGISSYAAKKVIDIIDAV
**ACC_6**	STVATIISLIGVIVGVGGITAGLVTTAKAMIKKYGKKYATMWLASMLGISTVAAKKVIDIIDTA
**ACC_7**	SSIYAIIGLIAAIAGGGGIGIGILFTAKALIKKFGKKYAAAWLASTLGISTYAAKKAIDLISAA
**ACC_8**	STVTTIISIITAVIGAGAITAGLVAVAKKMIKKYGKRYATMWLASMLGISKHAAEKVIDIIDTF
**ACC_9**	STIWSIVGIVAAVVGTGAIGVGILATAKTLVKKYGKAWAAAWLTSTLGISAYAAKKIIDIISTA
**ACC_10**	SAVATIIALVTAVVGGGLITAGIVATAKSLIKKYGAKYAAAWLTANLGISSYAAKKVIDIINTG
**ACC_11**	STVTTIISLVGLLVGYGVFTTALVATAKTVISKYGKKYAVTWLAANLGISTAVATKIITIIDTY
**ACC_12**	SNIALIVSLLAVIFGAGVITTGLVITAKYVIRRFGKKYAIAWLAANLGITTAAATRAIQIIDTA

Table S2. Origin, growth media, and incubation conditions of the bacterial strains used in this study.

Strain[a]	Origin[b]	Growth medium	Incubation conditions
Pediococcus damnosus CECT 4797	CECT	MRS	32 °C/ Aerobiosis
Lactococcus garvieae 5806	DNBTA	MRS	32 °C/ Aerobiosis
Enterococcus faecium ER46	VISAVET	MRS	37 °C/ Aerobiosis
Listeria monocytogenes CECT 4032	CECT	BHI	37 °C/ Aerobiosis
Staphylococcus aureus ZTA11/00117ST	VISAVET	BHI	37 °C/ Aerobiosis
Streptococcus suis C2969/03	VISAVET	BHI	37 °C/ Aerobiosis
Bacillus cereus ICM17/00252	VISAVET	BHI	37 °C/ Aerobiosis
Erysipelothrix rhusiopathiae ICM21/01900	VISAVET	BHI	37 °C/ Aerobiosis
Streptococcus agalactiae DICM11/00863	VISAVET	BHI	37 °C/ Aerobiosis
Paenibacillus larvae DB25	DNBTA	BHI	37 °C/ Aerobiosis
Clostridium perfringens DICM15/00067-5A	VISAVET	BHI	37 °C/ Anaerobiosis
Escherichia coli DH5α	Thermofisher	LB	37 °C/Agitation
Escherichia coli BL21 (DE3)	Thermofisher	LB	37 °C/Agitation

[a] Strains were used as indicator microorganisms, with the exception of *E. coli* BL21 (DE3), which was used for the *in vivo* production and purification of circular bacteriocins.

[b] DNBTA: Departamento de Nutrición, Bromatología y Tecnología de los Alimentos, Facultad de Veterinaria, Universidad Complutense de Madrid (UCM), Madrid, (Spain). VISAVET: Centro de Vigilancia Sanitaria Veterinaria, Universidad Complutense de Madrid (UCM), Madrid, (Spain). CECT: Colección Española de Cultivos Tipo, Valencia, (Spain).

Table S3. Amino acid sequences and physicochemical properties of the circular bacteriocin variants analyzed in this study.

Bacteriocin variant	Species origin	Percent Identity[a]	Length (aa)	Molecular mass (Da)	Theoretical pI	Net charge	Aliphatic index	GRAVY[b]	Mature amino acid sequence
AS-48_1	*Enterococcus faecalis*	100%	70	7150	10.09	6	117.14	0.539	MAKEFGIPAAVAGTVLNVVEAGGWVTTIVSILTAVGSGGLSLLAAAGRESIKAYLKKEIKKKGKRAVIAW
AS-48_2	*Listeria innocua, L. monocytogenes*	81%	70	7117	10.17	7	124.14	0.551	LAKEFGIPGGVAATVLNIVEAGGWATTILSILTAVGSGGLSLIAAAGKESIKVYLKKQIKKKGRKAVIAW
AS-48_3	*Bacillus safensis, B. pumilus*	80%	70	7087	10.00	5	125.43	0.579	LAKEFGIPGSVAAVVLNVVEAGGAVTTIVSILTAVGSGGLSLIAAAGKETIRQYLKNEIKKKGRKAVIAW
AS-48_4	*Paenibacillus larvae*	74%	70	7127	10.38	6	131.14	0.661	LAKEFGIPASIAAVVLNIVNAGGAVTTIVSILLAVGSGGLSLLAAAGKETIRQFLKKKIQEKGKRATIAW
AS-48_5	*Micrococcus* sp.	63%	70	7072	9.87	4	118.71	0.510	LAKEFGIPGGVASTVVWLAEAGGAAATIVGILAALGTGGLSLIAAAGKETIRQYLRKKIEEKGRKAVIAW
AS-48_6	*Paraclostridium sordellii*	59%	70	7362	10.66	7	113.14	0.386	LVKEFGIPAAGASGLLWYLDAGSSAATIVGFLTGLASGGLSLIYAAGRQTIKAYLRREIRKRGRKAVIAW
AS-48_7	*Geobacillus vulcani*	59%	70	7322	9.23	2	110.43	0.574	LAKEFGVPAWAAAWILTALDASTTVTAIVSFLTALGTGGLSLIAAAGTMGIKKYLKEELEKRGKQAFIAW
AS-48_8	*Paenibacillus larvae*	57%	70	7185	9.52	3	99.29	0.633	MVKEFGVPVAVATTVAKIMEAADWLITGAMILAAIGTGGASLLAAAGKESLKAFLKKKYGEMGARAFTAW
AS-48_9	*Thermoanaerobacterales*	53%	70	7416	10.09	6	124.14	0.680	LMKEFGLPSVAAYTILYYLNVGSTVTTIISILTGLATGGLSLIAAAGKLSIKAFLNKKKRELGTRAFVAW
AS-48_10	*Streptococcaceae*	43%	70	7017	9.82	3	128.43	0.881	LAKEFAIPSTAAVIAVDVIDNGMTLVSVAAVITAIGTGGASLVAAAEGQALRAFIKSQIRKIGKKAVIAW
GML_1	*Lactococcus garvieae*	100%	60	6007	10.13	5	115.83	0.887	LVATGMAAGVAKTIVNAVSAGMDIATALSLFSGAFTAAGGIMALIKKYAQKKLWKQLIAA
GML_ 2	*L. muris L. ileimucosae L. garvieae*	65%	60	6422	11.58	6	101.00	0.687	LVATGMSYTMARGIANAVSMGMNIWTAVSLFSGAFTGVAGIVALLRKYAQRKLWQRFIAA
GML_ 3	*Paenibacillus larvae*	47%	61	6086	9.53	2	121.80	0.913	LVAAGIGAGNAVAIINAIDNGMNIVTALSMFAGVAGAGAFVLANGLKWITKWAGKQTIINY
GML_ 4	*Lactococcus raffinolactis*	46%	61	6092	9.53	2	117.05	0.872	LVATGMGAGTAASVVDAIFGGLTIASALSLYAGLAGGAAWFLENGLKTLIKWAGKRTIISW
GML_ 5	*Thermoanaerobacterales*	76%	61	6190	6.18	0	120.33	0.844	LVATGMAAGTASTIVNAILNGMDIAAALSLYAGLAGGAAWFLEKGLTSLIRWAGRETIISW
ACC_1	*Bacillus amyloliquefaciens*	100%	64	6382	9.82	5	125.47	0.850	LASTLGISTAAAKKAIDIIDAASTIASIISLIGIVTGAGAISYAIVATAKTMIKKYGKKYAAAW
ACC_2	*Paenibacillus larvae*	98%	64	6368	9.82	5	123.91	0.845	LASTLGISTAAAKKAIDIIDAASTIASIISLIGIVTGAGAVSYAIVATAKTMIKKYGKKYAAAW
ACC_3	*Bacillus subtilis*	97%	64	6354	9.82	5	123.91	0.844	LASTLGISTAAAKKAIDIIDAASTVASIISLIGIVTGAGAISYAIVATAKSMIKKYGKKYAAAW
ACC_4	*Heyndrickxia coagulans*	94%	64	6354	9.82	5	122.34	0.841	LASTLGISTAAAKKAIDIIDTASTIASIISLIGVVTGAGAISYAVVATAKAMIKKYGKKYAAAW
ACC_5	*Clostridium* sp.	81%	64	6423	9.92	6	126.87	0.863	LASTLGISSYAAKKVIDIIDAVSTVATIISLATAVIGAGAISAAIVATAKKMIKKYGKKYAAAW
ACC_6	*Garciella nitratireducens*	70%	64	6508	9.90	5	131.09	0.970	LASMLGISTVAAKKVIDIIDTASTVATIISLIGVIVGVGGITAGLVTTAKAMIKKYGKKYATMW
ACC_7	*Sporolactobacillus vineae*	67%	64	6338	10.00	6	133.12	1.002	LASTLGISTYAAKKAIDLISAASSIYAIIGLIAAIAGGGGIGIGILFTAKALIKKFGKKYAAAW
ACC_8	*Marinitoga piezophila*	64%	64	6735	9.90	5	123.59	0.770	LASMLGISKHAAEKVIDIIDTFSTVTTIISIITAVIGAGAITAGLVAVAKKMIKKYGKRYATMW
ACC_9	*Helcococcus sp.*	64%	64	6415	10.00	5	132.81	1.014	LTSTLGISAYAAKKIIDIISTASTIWSIVGIVAAVVGTGAIGVGILATAKTLVKKYGKAWAAAW
ACC_10	*E. faecium, E. thailandicus*	61%	64	6317	9.90	5	134.38	0.953	LTANLGISSYAAKKVIDIINTGSAVATIIALVTAVVGGGLITAGIVATAKSLIKKYGAKYAAAW
ACC_11	*Kurthia sibirica*	61%	64	6649	9.70	4	134.06	0.991	LAANLGISTAVATKIITIIDTYSTVTTIISLVGLLVGYGVFTTALVATAKTVISKYGKKYAVTW
ACC_12	*Chloroflexota bacterium*	58%	64	6642	10.55	5	146.56	1.111	LAANLGITTAAATRAIQIIDTASNIALIVSLLAVIFGAGVITTGLVITAKYVIRRFGKKYAIAW

[a] Percent Identity relative to the reference variant (_1) of each bacteriocin group (query coverage varies among sequences).

[b] The grand average of hydropathicity (GRAVY) is a numerical index representing the overall hydrophobicity or hydrophilicity of a protein. GRAVY values > 0 indicate a predominantly hydrophobic protein, whereas GRAVY values < 0 indicate a predominantly hydrophilic protein.
